# Effects of Heat Stress on Breast Muscle Metabolomics and Lipid Metabolism Related Genes in Growing Broilers

**DOI:** 10.3390/ani14030430

**Published:** 2024-01-29

**Authors:** Xiumei Li, Xin Zhao, Miao Yu, Minhong Zhang, Jinghai Feng

**Affiliations:** State Key Laboratory of Animal Nutrition, Institute of Animal Sciences, Chinese Academy of Agricultural Sciences, Beijing 100193, China; llxiumei93@163.com (X.L.); zhaoxin09@caas.cn (X.Z.); ym161129@163.com (M.Y.); fjh6289@126.com (J.F.)

**Keywords:** chicken, high temperature, lipid regulatory factors, skeletal muscle, metabolomics

## Abstract

**Simple Summary:**

This paper used a metabolomics approach to study the effects of heat stress on general muscle metabolism. Heat stress led to disorders in breast muscle lipid metabolism in broilers. The gene for peroxisome proliferator-activated receptor alpha might be the key gene in the mechanism of lipid metabolism that HS induced in the breast muscles of the broilers. These findings provide new insights for the development of the modern broiler industry under heat stress.

**Abstract:**

With global warming and worsening climatic conditions, heat stress (HS) has become a significant challenge affecting the development of poultry production. In this study, we aimed to determine the effects of HS on breast muscle metabolomics and lipid metabolism-related genes in growing broilers. One hundred twenty 29-day-old Arbor Acres broilers were randomly divided into normal temperature (NT; 21 ± 1 °C) and heat stress (HS; 31 ± 1 °C) groups, with six replicates (ten birds in each replicate) in each group, raised for 14 days in two environment chambers at 60 ± 7% relative humidity. Compared with the broilers in the NT group, the average daily food intake, average daily gain and breast muscle yield in the HS group were significantly lower (*p* < 0.05). The feed conversion ratio was significantly higher in the HS group (*p* < 0.05). The concentrations of serum corticosterone, free fatty acids and cholesterol and the percentage of abdominal fat of broilers in the HS group were significantly higher (*p* < 0.05) than the values of the broilers in the NT group. Untargeted breast muscle metabolome analysis revealed 14 upregulated differential metabolites, including glycerophosphocholine, and 27 downregulated differential metabolites, including taurine, in the HS group compared to the NT group; the HS group also displayed significant effects on six metabolic pathways compared to the NT group (*p* < 0.05). The mRNA expression levels of peroxisome proliferator-activated receptor gamma coactivator-1-alpha, peroxisome proliferator-activated receptor alpha (*PPARα*) and ATP-binding cassette transporter A1 in the liver and breast muscles were significantly decreased in the HS group compared with the NT group (*p* < 0.05). The collective findings reveal that HS can cause disorders in breast muscle lipid metabolism in broilers. The *PPARα* gene might be the key gene in the mechanism of the lipid metabolism that is induced by HS in breast muscle of broilers. These findings provide novel insights into the effects of HS on chicken growth.

## 1. Introduction

Heat stress (HS) is one of the most important problems in global poultry production [1], and can cause significant economic losses [2]. The costs of rearing under adequate conditions in the poultry farming industry are expected to increase as the global temperature continues to rise in the coming decades [3]. HS negatively affects the animals’ health and can lead to hyperthermia, which can produce physiological and pathological changes [4,5]. Numerous studies have shown that HS damages the wellbeing and growth performance of broilers; for example, broilers have reduced feed intake and the growth rate of broilers under HS [3,6,7,8]. HS has a significant effect on breast muscle growth and can significantly reduce breast muscle yield [9,10,11]. When broilers experience HS, more energy is used for fat storage [12,13], and excessive fat accumulation in broilers damages the breeding and processing of chicken meat [13]. It has previously been reported that HS impairs the lipid metabolism in broilers by reducing protein deposition and promoting fat synthesis, which increases the production rates of abdominal, subcutaneous and intramuscular fat [14,15]. At present, poultry meat production is increasing rapidly [16]. Breast muscle is a highly metabolic tissue that plays a crucial role in promoting metabolism and nutrient storage in the body. Almost 50% of the total body mass comprises skeletal muscle, which exhibits strong metabolic activity and is the largest source of protein in the body [17]. However, few studies have addressed breast muscle metabolomics in broiler chickens.

Lipid is one of the important biological macromolecules in animals, which has a variety of biological functions, including energy storage, signal recognition and immunity [18]. A study has shown that lipid metabolism is closely related to the maintenance of dynamic energy balance and the physiological function of broilers [19]. Fat is absorbed and metabolized. The biochemical processes involved in the metabolism of fat in vivo include the metabolism of triglycerides, phospholipids, cholesterol and plasma lipoproteins, with many genes being involved [20]. ATP-binding cassette transporters are a superfamily of conserved membrane proteins responsible for lipid transport, and the reports suggest that cholesterol synthesis is upregulated under HS [21]. Peroxisome proliferator-activated receptor gamma coactivator-1-alpha (*PGC-1α*) and peroxisome proliferator-activated receptor alpha (*PPARα*) gene activities are related to the regulation of skeletal muscle metabolism, which could drive the transcription of a vast gene network involving many aspects of energy homeostasis, including glucose utilization and fatty acid oxidation [22].

Metabolomics can quantitatively measure small molecule metabolites in biological samples [23]. Liquid chromatography—mass spectrometry (LC-MS) has been widely used in metabolomics studies because of its high detection sensitivity and sample derivatization [14]. Furthermore, LC-MS can detect intact metabolites without chemical modifications [24]. Therefore, we used untargeted metabolomics to reveal the effect of HS on breast muscle metabolism and aimed to analyze the genes related to lipid metabolism in broilers under HS. The findings are fundamentally important for studies of the characteristics of lipid metabolism in growing broilers under HS.

## 2. Materials and Methods

### 2.1. Birds and Treatments

A total of 120 29-day-old Arbor Acres (AA) male broilers with similar body weight (1150 ± 70 g) were chosen. They were randomly assigned to the normal temperature (21 ± 1 °C, NT) or HS (31 ± 1 °C) group for 2 weeks, until they were 42-days-of-age. They were housed in six one-tier cages (10 birds per cage; each cage served as a replicate) in two environmentally controlled chambers. Other than the temperature, the chambers were identical (60 ± 7% relative humidity, 24-h light and wind velocity < 0.5 m/s). The cage size was 0.80 m length × 0.80 m width × 0.40 m height. All broilers had ad libitum access to water and to a standard corn-soybean meal diet, consistent with the NRC requirements for AA broilers.

### 2.2. Sample Collection and Chemical Analysis

For each treatment group, 12 broilers were randomly chosen (two birds from each replicate) at 42 days of age and euthanized through carbon dioxide inhalation. 

#### 2.2.1. Growth Performance Measurement

The growth performance of the broilers from 29 to 42-days-of-age was assessed by determining the average daily food intake (ADFI), average daily gain (ADG) and feed conversion ratio (FCR) on a replicate basis. The left and right sides of the breast muscles of the broilers were weighed and divided by body weight to calculate the breast muscle yield.

#### 2.2.2. Lipid Parameters

Abdominal fat was weighed to calculate the percentage of abdominal fat. Blood (5 mL; brachial vein) was collected and centrifuged at 3000× *g* for 15 min at 4 °C to separate the plasma and serum. The latter was stored at −20 °C for subsequent analysis of corticosterone, free fatty acids and cholesterol using a fully automatic biochemical analyzer (Hitachi 7600, Hitachi, Japan) according to the manufacturer’s instructions.

#### 2.2.3. LC-MS 

LC-MS of the breast muscle samples was performed as previously described [25] using a mobile phase elution gradient, 2 μL injection volume, holding temperature of 40 °C and flow rate of 0.40 mL/min. An Acquity UPLC HSS T3 column (100 mm × 2.1 mm, 1.7 µm; Waters, Milford, MA, USA) was used. Quality control (QC) was performed before, during and after detection by mixing equal amounts of the sample. The pre-injection QC was performed to monitor the instrument status and equilibrium chromatography—mass spectrometry system before injection. QC during sample detection evaluated the stability of the system and allowed sample correlation analysis throughout the experiment. Finally, QC after sample detection validated the qualitative analysis of metabolites through secondary mass spectrometry.

#### 2.2.4. Expression of Genes for Regulatory Factors

Approximately 10 g of the left side of each breast muscle was collected, frozen using liquid nitrogen and stored until analysis. Total RNA was extracted from each frozen breast muscle sample using TRIzol reagent (Invitrogen, Carlsbad, CA, USA), and real-time PCR was conducted using a LightCycler 96 system (LightCycler 96 system, Roche, Basel, Switzerland), according to the manufacturers’ instructions. The mRNA levels of ATP-binding cassette transporter A1 (*ABCA1*), ATP-binding cassette transporter G1 (*ABCG1*), *PPARα* and *PCG-1α* in the breast muscle and liver tissues were measured. The expression of the target genes relative to the house keeping gene (*β-actin*) in the samples were calculated using the 2^−ΔΔCt^ method. The primers for the target and *β-actin* genes are shown in Table 1. 

### 2.3. Statistical Analysis

One-way analysis of variance was used to analyze the data using the SAS 9.2 software (SAS Institute Inc., Cary, NC, USA). One replicate was used as the experimental unit to analyze ADG, ADFI and FCR. The mean of two sampled birds per replicate was used as the experimental unit to analyze other parameters (*n* = 6). All data are expressed as the mean ± SD. Statistical significance was set at *p* < 0.05. The metabolic data were analyzed using orthogonal projection latent structure discriminant analysis (OPLS-DA). The variable importance in the projection (VIP) value of the OPLS-DA model (VIP ≥ 1) and independent sample *t* test (*p* < 0.05) were used to screen differential metabolites. Differential metabolites in the HS and NT groups were mapped to the Kyoto Encyclopedia of Genes and Genomes (KEGG) Identifier using the MetaboAnalyst (4.0) online software. Pathway analysis was performed. The house mouse (Mus musculus) was selected as the model organism. Significant pathways (*p* < 0.05) were identified through KEGG analysis.

## 3. Results

### 3.1. Growth Performance

All of the broilers were healthy during the experimental period. As shown in Table 2, compared to the broilers in the NT group, the HS groups displayed significantly lower ADFI, ADG and breast muscle yield values (*p <* 0.05) and significantly higher FCR (*p <* 0.05).

### 3.2. Lipid Parameters

As shown in Table 3, the concentrations of serum corticosterone, free fatty acid, cholesterol and the percentage of abdominal fat of broilers in the HS group were significantly higher (*p* < 0.05) than those of the broilers in the NT group.

### 3.3. Metabolite Analysis

**Figure 1:**
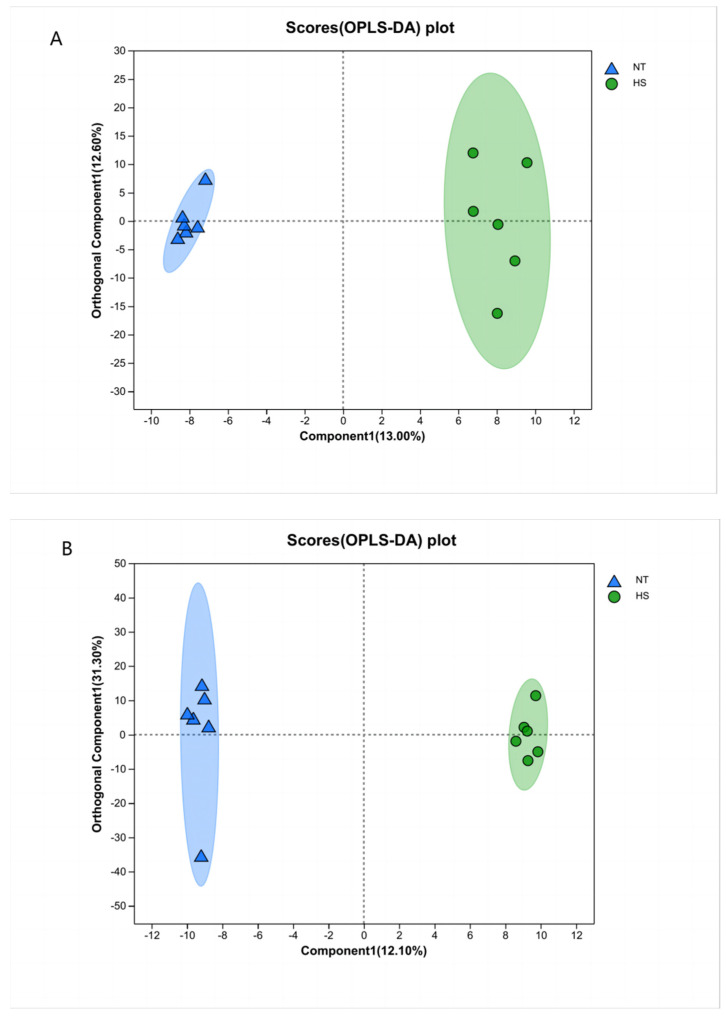
OPLS-DA dimension reduction analysis of metabolites in NT and HS groups. Positive mode of scores plot (**A**), negative mode of scores plot (**B**). (Blue) NT, normal temperature; (Green) HS, heat stress; OPLS-DA, orthogonal projection latent structure discriminant analysis; *n* = 6.

The scores (OPLS-DA) plots are shown in Figure 1. There is an obvious separating trend between the two groups. In the model of the positive mode of the scores plot, R^2^X = 0.256, R^2^Y = 0.990, Q^2^ = 0.470; in the model of the negative mode of the scores plot, R^2^X = 0.556, R^2^Y = 0.998, Q^2^ = 0.554.

According to the OPLS-DA model (VIP ≥ 1) and independent sample *t* test (*p* < 0.05), 41 differential metabolites were screened between the two groups. The screening results of the differential metabolites are shown in Table 4. Compared with the NT group, there are 14 up-regulated differential metabolites and 27 down-regulated differential metabolites in the HS group. Compared with the NT group, the concentrations of phosphatidyl ethanolamine (PE) (P-16:0/20:4(6E,8Z,11Z,14Z)(5OH[S])), PE (NMe(18:0/22:5(4Z,7Z,10Z,13Z,16Z))), phosphatidylcholine (PC) (16:0/22:6(4Z,7Z,10Z,13Z,16Z,19Z)), sphingomyelin (SM) (d18:1/16:0), alanyl-dl-leucine, alanyl-tryptophan, linoelaidyl carnitine, 1,2-dipalmitoyl-sn-glycero-3-PC, octadecenoylcarnitine, spermidine, alanyl-dl-phenylalanine, decanoyl-L-carnitine, 3-hydroxyhexadecanoyl carnitine, 25-Hydroxyvitamin D3-26,23-lactol, rockogenin, pipericine, 2-Methylbutyroylcarnitine, Cis- and trans-L-Mercapto-p-menthan-3-one, histamine, alanyl-tyrosine, palmitoyl sphingomyelin, alanyl-arginine, 3-Phenylpropyl glucosinolate, nicotinamide adenine dinucleotide, phenylalanyl-gamma-glutamate, 9(Z),11(E)-conjugated linoleic acid and taurine were lower in the HS group (VIP > 1 and *p* < 0.05), while PE (P 18:1(9Z)/16:1(9Z)), PE (18:2(9Z,12Z)/P-16:0), PE (18:2(9Z,12Z)/18:0), PE (15:0/22:2(13Z,16Z)), PC (18:2(9Z,12Z)/P-16:0), glycerophosphocholine, persicaxanthin, delphinidin 3-rutinoside, D-glucuronic acid, fructosamine, 1-(11Z,14Z-eicosadienoyl)-glycero-3-phosphate, 1-arachidonoylglycerophosphoinositol, N-(1-Deoxy-1-fructosyl) glycine and sedoheptulose 1-phosphate (VIP > 1 and *p* < 0.05) were higher in the HS group.

### 3.4. Metabolite Pathways and Expression of Genes for Regulatory Factors

**Figure 2:**
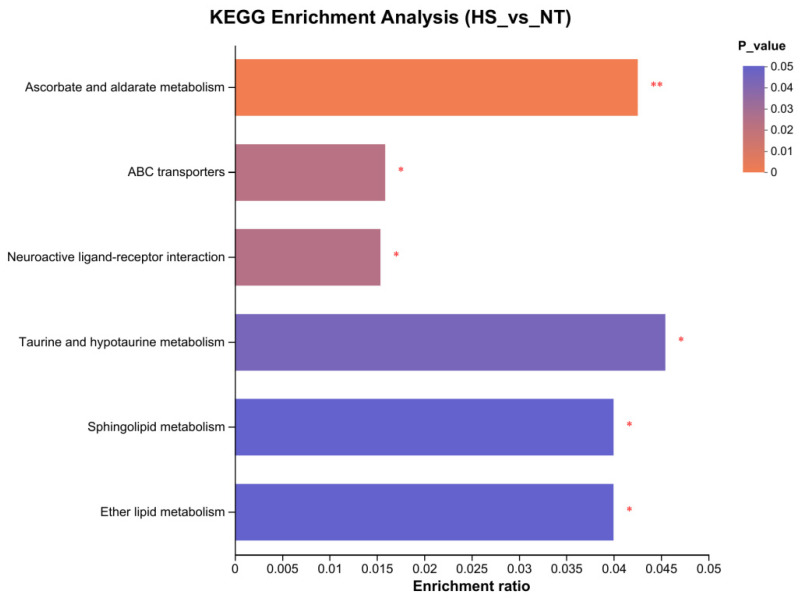
KEGG enrichment analysis of metabolites in the NT and HS groups. The ordinate represents the pathway name, and the abscissa represents the enrichment rate, which represents the ratio of the number of metabolites enriched in the pathway (Metabolite number) to the number of metabolites annotated to the pathway (Background number). The greater the ratio, the greater the degree of enrichment, the color gradient of the column indicates the significance of the enrichment. The darker the default color, the more significant enrichment of the KEGG term, where *p*-value < 0.01 is marked as **, *p*-value < 0.05 marked with *. NT, normal temperature; HS, heat stress; KEEG, Kyoto Encyclopedia of Genes and Genomes.

The pathway analysis revealed that HS had a significant effect on six metabolic pathways compared to the NT group: ascorbate and aldarate metabolism, taurine and hypotaurine metabolism, ether lipid metabolism, sphingolipid metabolism, ABC transporters and neuroactive ligand-receptor interactions (Figure 2; *p* < 0.05).

**Figure 3:**
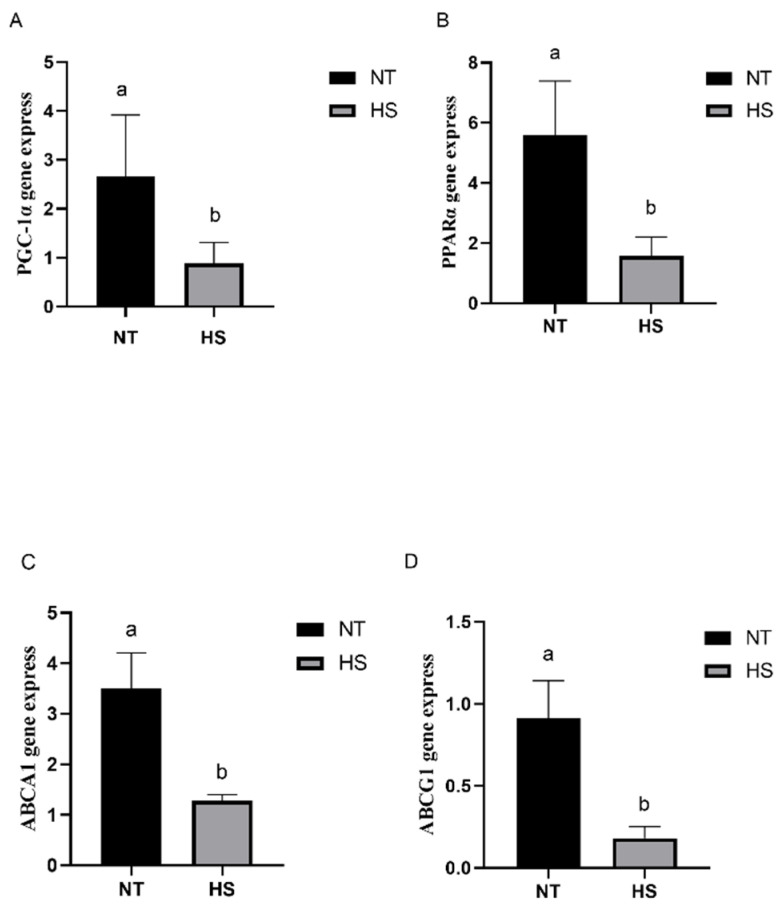
Effects of heat stress on the mRNA expression levels of related regulatory factors in liver of broilers. *PGC-1α* (**A**), *PPARα* (**B**), *ABCA1* (**C**) and *ABCG1* (**D**) gene express. *PGC-1α*, peroxisome proliferator-activated receptor gamma coactivator-1-alpha; *PPARα*, peroxisome proliferator-activated receptor alpha; *ABCA1*, ATP-binding cassette transporter A1; *ABCG1*, ATP-binding cassette transporter G1; NT, normal temperature; HS, heat stress. Each bar presents means ± SD (*n* = 6). ^a,b^ Values with different superscripts differ significantly at *p* < 0.05.

**Figure 4:**
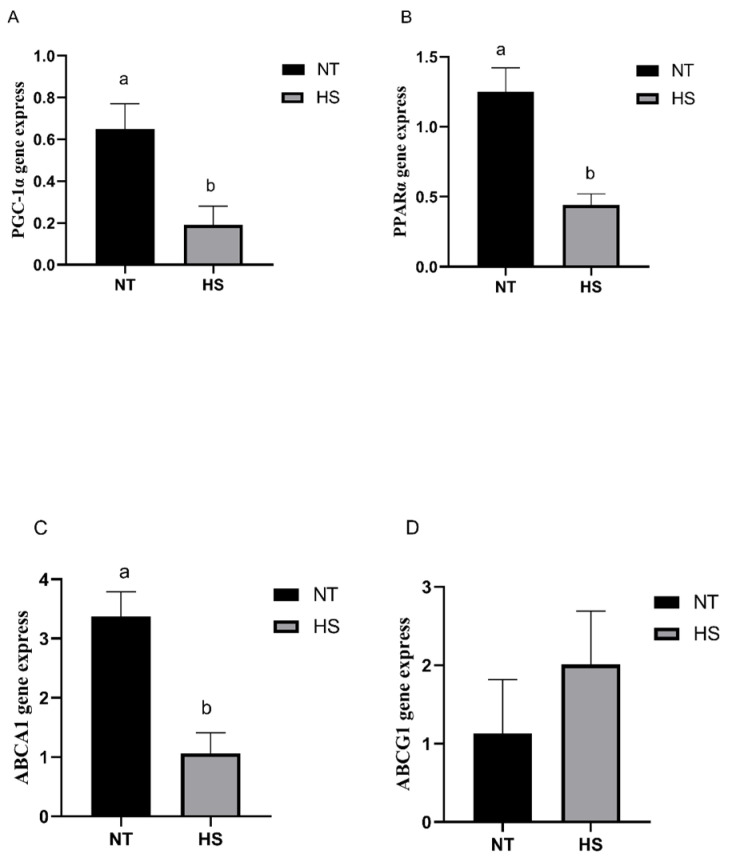
Effects of heat stress on the mRNA expression levels of related regulatory factors in breast muscle of broilers. *PGC-1α* (**A**), *PPARα* (**B**), *ABCA1* (**C**) and *ABCG1* (**D**) gene express. *PGC-1α*, peroxisome proliferator-activated receptor gamma coactivator-1-alpha; *PPARα*, peroxisome proliferator-activated receptor alpha; *ABCA1*, ATP-binding cassette transporter A1; *ABCG1*, ATP-binding cassette transporter G1; NT, normal temperature; HS, heat stress. Each bar presents means ± SD (*n* = 6). ^a,b^ Values with different superscripts differ significantly at *p* < 0.05.

The gene expression of the breast muscle- and liver-related regulatory factors in the broilers exposed to constant HS was determined. The mRNA expression levels of *PGC-1α*, *PPARα*, *ABCA1* and *ABCG1* in the liver and breast muscle are presented in Figure 3 and Figure 4. The HS group showed significantly decreased mRNA expression levels of *PGC-1α*, *PPARα*, *ABCA1* and *ABCG1* in the liver compared with the NT group (Figure 3; *p* < 0.05). In addition, the HS group showed significantly decreased mRNA expression levels of *PGC-1α*, *PPARα* and *ABCA1* in the breast muscle compared with the NT group (Figure 4; *p* < 0.05). In contrast, there was no significant effect on the mRNA expression level of *ABCG1* in the two groups (Figure 4; *p* > 0.05).

## 4. Discussion

An increase in ambient temperature causes the body temperature to exceed the normal range (thermal neutral zone), causing normal physiological functions in the body to be affected, and this includes cell damage [26]. Animals adopt certain physiological and metabolic adjustments to maintain their body temperature when exposed to high environmental temperatures, at the expense of growth and reproduction [27]. Many experiments have shown that HS can reduce feed intake, feed utilization efficiency and carcass quality, and adversely affects broiler growth [7,28,29]. Our results are consistent with the previous findings. In the present study, the HS group had significantly reduced growth performance and breast muscle yield compared with the NT group. Piestun et al. (2017) [30] elucidated that continuous heat exposure leads to impaired myogenicity of the satellite cells and increased fat deposition. HS has previously been reported to significantly reduce breast muscle weight in broiler chickens [31]. Moreover, HS impairs the meat quality by causing mitochondrial malfunction and affects aerobic metabolism, resulting in increased glycolysis and intramuscular fat deposition [32]. However, the data on breast muscle metabolomics in heat-stressed broilers are still limited, and this lack was addressed in the present study.

HS induces changes in the activity of the chicken neuroendocrine system, such as the activation of the hypothalamic-pituitary-adrenal axis and the increased production of concentrations of corticosterone, which is a reliable indicator of HS in chickens [33]. In the present study, the serum corticosterone concentration of the broilers in the HS group was significantly higher than that of the broilers in the NT group, which can be taken as a sign of HS in chickens. This finding indicates that the broilers in the heat-treatment group were already under HS, which raised another problem. Broilers reportedly exhibit excessive fat deposition when exposed to high temperatures [34]. After fat absorption, the biochemical processes of metabolism in vivo include the metabolism of triglycerides, phospholipids, cholesterol and plasma lipoproteins [20]. Free fatty acids and cholesterol play important roles as building blocks of animal cell membranes and are precursors for many important lipid molecules that are harmful to animals when present in excess [35]. The maintenance of lipid homeostasis is important for growth, and cholesterol and fatty acids levels must be tightly controlled. In the present study, the HS group of broilers had a significantly increased percentage of abdominal fat and concentrations of serum free fatty acids and cholesterol, indicating that HS disrupts the lipid metabolism balance. Similarly, Luo et al. (2018) [36] demonstrated that HS increased the serum triglyceride and total cholesterol concentrations in AA broilers. Skeletal muscle accounts for almost all of the edible parts of the broiler body and is the tissue that is responsible for 70–80% of glucose conversion after the ingestion of carbohydrates [37], which is important for broiler metabolism. Previous studies have focused on HS-disordered serum metabolism in broiler chickens. Zhang et al. (2018) [38] observed that a 32 °C feeding environment reduced the levels of stearic, arachidonic, palmitic and oleic acids in AA broilers. Guo et al. (2020) [39] reported that HS alters the serum lipid metabolite concentrations in indigenous slow-growing broiler chickens. However, there are few reports on the effects of HS on breast muscle metabolomics in broiler chickens. In the present study, the LC-MS analysis of the breast muscle metabolites of broilers revealed that HS altered the levels of 41 breast muscle metabolites, including lipid metabolites PC, PE, SM, glycerophosphocholine, etc., and six KEGG metabolites-enriched pathways closely related to energy metabolism in the body. These results suggest that HS induces breast muscle metabolic disorders, particularly lipid metabolism, in broilers. PC and PE are glycerol phospholipids that are important biofilm components. Phospholipids are the main lipid carriers in plasma and can regulate lipid metabolism [40]. SM is an important phosphate in biological membranes and a vital marker of dyslipidemia that acts on the oxidation of low-density proteins [41]. These observations are consistent with those of a previous study [38,39]. We assumed that HS reduces phospholipid levels and damaged cell membranes, probably because of a reduction in ADFI during HS, resulting in energy metabolism disorders in broilers.

In addition, our metabolome data revealed that HS can significantly affect the ABC transporters metabolic pathway. This pathway involves multiple ATP-binding cassette transporter proteins, including *ABCA1* and *ABCG1* [42]. *ABCA1* and *ABCG1* are expressed in multiple tissues and act synergistically to pump specific substrates across membranes [43]. Several studies have reported that the role of *ABCA1* is to promote the outflow of cholesterol and choline phospholipids into lipid-poor apolipoproteins [44,45,46]. *ABCG1* is a critical gene involved in lipid homeostasis in vivo and promotes the efflux of cholesterol from macrophages to high-density lipoproteins [47]. *PPARα* regulates the expression of genes involved in peroxisome and mitochondrial oxidation, and can regulate the expression of genes related to fatty acid synthesis, oxidation, transport and storage, can reduce lipid deposition and is closely related to the regulation of glycolipid metabolism and energy balance as a predominant regulator of lipid homeostasis [48,49]. *PGC-1α* is an inducible co-regulator of nuclear receptors and is involved in a wide variety of biological responses [50]. *PGC-1α* is a coactivator of *PPAR-γ*, which is a key factor in mitochondrial biogenesis [51]. It is a key factor in mitochondrial biosynthesis and is highly expressed in skeletal muscles, especially in oxidized muscle fibers [52]. The study of mice has shown *PGC-1α* upregulation apparently modulates transcriptional reprogramming to increase the mitochondrial mass and fatty acid oxidative metabolism in muscle [53]. *PGC-1α*, *PPARα*, *ABCA1* and *ABCG1* are involved in the lipid regulation of tissues and organs. A previous study reported that *PPARα* activator induces the expression of the gene encoding *ABCA1* [54]. In the present study, HS significantly decreased the mRNA expression levels of *PGC-1α*, *PPARα* and *ABCA1* in the liver and breast muscle and decreased the mRNA expression level of *ABCG1* in the liver. These findings indicate that HS affects the lipid metabolites in the breast muscle and liver of broilers. The results further suggest that these genes are involved in lipid metabolism, which could explain the increased percentage of abdominal fat and concentrations of serum free fatty acids and cholesterol in the broilers exposed to HS. Numerous studies have shown that the liver is responsive to HS, which alters lipid metabolism [55,56].

## 5. Conclusions

In summary, the LC-MS-based breast muscle analysis and mRNA expression data of the genes related to lipid metabolism demonstrate that HS decreases growth performance and increases fat deposition. HS induces lipid metabolism disorders in breast muscle and the *PPARα* gene may be the key gene in the mechanism of lipid metabolism. 

## Figures and Tables

**Table 1:** Primers used for quantitative RT-PCR.

Primer Name ^1^	Primer Sequence ^2^ 5′-3′	Product Size (bp)	GenBank Accession Number
*β-actin*	F: CTGTGTTCCCATCTATCGT	270	NM_205518
	R: TCTTCTCTCTGTTGGCTTTG		
*ABCA1*	F: TCATCCACCGCCGCCACATT	223	NM_204145
	R: GGCTGAGGAAGGCACTGAAGTC		
*ABCG1*	F: AACCAGTGGCTTGGATAGTGC	298	XM_025145525
	R: CCTTACCAGTCGGCTGTTCTG		
*PPARα*	F: CAAACCAACCATCCTGACGAT	22	NM_001001464
	R: CTCCACTGTCACTCAGGTTTCCT		
*PGC-1α*	F: GGGACCGGTTTGAAGTTTTTG	110	NM_001006457
	R: GGCTCGTTTGACCTGCGTAA		

^1^ *β-actin*, beta-actin; *ABCA1*, ATP-binding cassette transporter A1; *ABCG1*, ATP-binding cassette transporter G1; *PPARα*, peroxisome proliferator-activated receptor alpha; *PGC-1α*, peroxisome proliferator-activated receptor gamma coactivator-1-alpha. ^2^ F, forward; R, reverse.

**Table 2:** Effects of heat stress on the growth performance of broilers ^1^.

Items	NT ^2^	HS ^2^	*p*-Value
ADFI (g)	129.40 ^a^ ± 2.39	97.21 ^b^ ± 4.22	<0.01
ADG (g)	76.81 ^a^ ± 2.39	56.37 ^b^ ± 3.15	<0.01
FCR	1.68 ^b^ ± 0.02	1.74 ^a^ ± 0.03	<0.01
Breast muscle yield	19.74 ^a^ ± 1.55	17.85 ^b^ ± 1.27	<0.01

ADFI = average daily feed intake; ADG = average daily gain; FCR = ADFI/ADG. ^1^ All the means are reported as the means ± SD (*n* = 6). ^2^ NT, normal temperature; HS, heat stress. ^a,b^ Values with different superscripts differ significantly at *p <* 0.05.

**Table 3:** Effects of heat stress on lipid parameters of broilers ^1^.

Items	NT ^2^	HS ^2^	*p*-Value
Cholesterol(mmol/L)	3.65 ^b^ ± 0.23	4.80 ^a^ ± 0.11	<0.01
Free fatty acids (μmol/L)	58.61 ^b^ ± 5.72	106.68 ^a^ ± 3.24	<0.01
Corticosterone(ng/mL)	10.74 ^b^ ± 1.71	15.78 ^a^ ± 0.51	<0.01
Percentage of abdominal fat (%)	0.51 ^b^ ± 0.20	0.77 ^a^ ± 0.20	<0.01

^1^ All the means are reported as the means ± SD (*n =* 6). ^2^ NT, normal temperature; HS, heat stress. ^a,b^ Values with different superscripts differ significantly at *p* < 0.05.

**Table 4:** Differential metabolites in breast muscle of broilers in the NT and HS groups ^1^.

Metabolites ^2^	VIP (HS&NT)	FC (HS/NT)	*p*-Value	Change
PE				
P-18:1(9Z)/16:1(9Z)	1.647	1.0298	0.0277	↑
P-16:0/20:4(6E,8Z,11Z,14Z)(5OH[S])	1.557	0.9679	0.0343	↓
18:2(9Z,12Z)/P-16:0	1.869	1.0429	0.0101	↑
18:2(9Z,12Z)/18:0	1.736	1.0362	0.0151	↑
15:0/22:2(13Z,16Z)	1.185	1.0129	0.0232	↑
NMe(18:0/22:5(4Z,7Z,10Z,13Z,16Z))	1.244	0.9826	0.0352	↓
PC				
18:2(9Z,12Z)/P-16:0	1.495	1.0231	0.0441	↑
16:0/22:6(4Z,7Z,10Z,13Z,16Z,19Z)	1.624	0.9703	0.0211	↓
SM(d18:1/16:0)	1.383	0.9835	0.0178	↓
Glycerophosphocholine	2.547	1.0800	0.0410	↑
Alanyl-dl-leucine	2.398	0.9357	0.0096	↓
Alanyl-tryptophan	2.698	0.9340	0.0008	↓
Linoelaidyl carnitine	3.730	0.8654	0.0277	↓
1,2-dipalmitoyl-sn-glycero-3-PC	1.569	0.9715	0.0175	↓
Octadecenoylcarnitine	3.729	0.8656	0.0259	↓
Spermidine	2.409	0.9283	0.0296	↓
Alanyl-dl-phenylalanine	2.703	0.9286	0.0019	↓
Decanoyl-L-carnitine	3.664	0.8284	0.0097	↓
3-hydroxyhexadecanoyl carnitine	3.641	0.8586	0.0108	↓
25-Hydroxyvitamin D3-26,23-lactol	2.689	0.9132	0.0394	↓
Rockogenin	3.520	0.8535	0.0104	↓
Persicaxanthin	2.482	1.0753	0.0159	↑
Pipericine	1.559	0.9581	0.0253	↓
2-Methylbutyroylcarnitine	2.277	0.9442	0.0260	↓
Cis- and trans-L-Mercapto-p-menthan-3-one	2.291	0.9244	0.0126	↓
Delphinidin 3-rutinoside	2.111	1.0522	0.0190	↑
Histamine	1.212	0.9763	0.0487	↓
Alanyl-tyrosine	2.143	0.9522	0.0085	↓
D-Glucuronic acid	1.734	1.0359	0.0194	↑
Palmitoyl sphingomyelin	1.371	0.9813	0.0159	↓
Alanyl-arginine	3.389	0.8285	0.0461	↓
Fructosamine	2.649	1.0851	0.0029	↑
3-Phenylpropyl glucosinolate	3.321	0.8777	0.0105	↓
Nicotinamide adenine dinucleotide	1.848	0.9639	0.0108	↓
Phenylalanyl-Gamma-glutamate	2.449	0.9334	0.0027	↓
1-(11Z,14Z-eicosadienoyl)-glycero-3-phosphate	1.819	1.0395	0.0044	↑
1-Arachidonoylglycerophosphoinositol	1.991	1.0516	0.0342	↑
9(Z),11(E)-Conjugated Linoleic Acid	3.512	0.7954	0.0388	↓
N-(1-Deoxy-1-fructosyl) glycine	2.695	1.0924	0.0037	↑
Sedoheptulose 1-phosphate	1.666	1.0395	0.0278	↑
Taurine	2.683	0.9176	0.0309	↓

^1^ NT, normal temperature; HS, heat stress; VIP, variable importance in the projection; FC, fold change. ^2^ PE, phosphatidyl ethanolamine; PC, phosphatidylcholine; SM, sphingomyelin. ↑, up-regulated differential metabolites in the HS group compared with the NT group; ↓, down-regulated differential metabolites in the HS group compared with the NT group.

## Data Availability

Data are contained within the article.
